# Nutrimetabolomics: An Update on Analytical Approaches to Investigate the Role of Plant-Based Foods and Their Bioactive Compounds in Non-Communicable Chronic Diseases

**DOI:** 10.3390/ijms17122072

**Published:** 2016-12-09

**Authors:** Oscar Daniel Rangel-Huerta, Angel Gil

**Affiliations:** 1 Department of Biochemistry and Molecular Biology II, Institute of Nutrition and Food Technology “José Mataix”, Center for Biomedical Research, University of Granada, 18100 Granada, Spain; odrangel@ugr.es; 2 Centro de Investigación Biomédica en Red de Fisiopatología de la Obesidad y Nutrición, Ciberobn, 28029 Madrid, Spain

**Keywords:** bioactive compounds, metabolomics, non-communicable chronic diseases, nutrimetabolomics, plant-based food

## Abstract

Metabolomics is the study of low-weight molecules present in biological samples such as biofluids, tissue/cellular extracts, and culture media. Metabolomics research is increasing, and at the moment, it has several applications in the food science and nutrition fields. In the present review, we provide an update about the most frequently used methodologies and metabolomic platforms in these areas. Also, we discuss different metabolomic strategies regarding the discovery of new bioactive compounds (BACs) in plant-based foods. Furthermore, we review the existing literature related to the use of metabolomics to investigate the potential protective role of BACs in the prevention and treatment of non-communicable chronic diseases, namely cardiovascular disease, diabetes, and cancer.

## 1. Introduction

Non-communicable chronic diseases (NCCDs) are highly prevalent in both developing and developed countries [1]. The prevalence of NCCDs, which mainly includes heart disease, stroke, cancer, diabetes, and chronic lung disease is increasing, and currently they are responsible for almost 70% of all deaths worldwide [1]. The rise of NCCDs is primarily associated with four major risk factors: tobacco use, physical inactivity, excessive alcohol intake, and unhealthy diet. Therefore, modifying lifestyles, including diet, may help to reduce the prevalence of NCCDs. In this regard, it is known that dietary habits are quite different around the world; nevertheless, certain dietary patterns are common worldwide, for example, the inclusion of fruits, vegetables, or other plant-based products to the diet [2].

Food bioactive compounds (BACs) are “extra nutritional” constituents that can be found in small quantities, particularly in plant-based products [3]. The growing body of scientific evidence indicates that several BACs play a beneficial role in the prevention of diseases [4,5]. Indeed, BACs that are present in plant-based products can increase the intake of ingredients reputed to have healthy benefits.

Nowadays, one of the main outcomes of modern nutrition is preventing NCCDs, characterizing the relationship between diet, lifestyle, and health at a molecular level, and identifying the potential healthy role of nutrients as well as BACs. Therefore, the novel “omics” sciences, which include genomics, transcriptomics, epigenomics, proteomics, and metabolomics, are helping to understand the mechanisms by which foods and their BACs may influence the cellular tissue and organ metabolism and how they may contribute to host health.

Particularly, metabolomics studies the global changes in the metabolites present in cells, tissues, organs, and organisms. The metabolites are low- and medium-weight molecules (<1500 Dalton) derived from the process known as metabolism (i.e., substrates and products from the enzymes) [6]. Several approaches indicate that the number of metabolites present in human beings are around 3000 and 20,000, whereas the number of genes is about 20,500, and the number of proteins are more than 100,000 [7].

The omics sciences, applied to nutrition and health, have the potential to identify health biomarkers, such as early biomarkers associated with different diseases and the modifications derived from food consumption [8,9]. Furthermore, omics tools may help to differentiate between responsive and non-responsive subjects to nutritional interventions, and also may help to discover new bioactive compounds from foods and their effect on the organism.

Metabolomics has shown different applications in food sciences. For example, the study of fruits, vegetables, and seeds have arisen in various fields, such as the clarification of developmental physiology, metabolic pathways, and genes involved in the improvement of fruit yield and quality [10,11,12,13]. Moreover, processed food products can be deliberately substituted partially or entirely, with similar, lower quality, and cheaper counterparts or unintentional errors can cause inadvertently mislabeled products. Thus, metabolomics may serve as a determinant tool regarding food authentication [14] and may contribute to the assessment of food safety, quality, and traceability [15,16].

In addition, the use of metabolomics in nutrition sciences, also known as nutrimetabolomics, may be an attractive instrument for the identification of how nutrients and food BAC can interact and modulate metabolic diseases [17]. The knowledge provided from this science may serve in developing accurate, personalized dietary treatments. Furthermore, nutrimetabolomics opens the door to a new age of research and development lines due to massive dataset generation. The use of nutrimetabolomics data in bioinformatics could allow research work in fields such as the determination of the nutritional status of individuals and populations or predicting and analyzing the responses to dietary interventions [18,19,20,21].

The objective of the present paper is to provide an update about the current knowledge of analytical technologies and approaches using metabolomics. Furthermore, it aims to review the current applications in food sciences and the existing evidence of nutrimetabolomics use, studying the effects of nutrients and certain BACs that are present in plant-based foods such as vegetables, whole grains, legumes, fruits, and plant extracts on NCCDs outcomes.

## 2. Methodological Approaches

The metabolome complexity depends on several factors, e.g., the inter-individual variation, the natural occurring dynamic of the compounds forming the metabolome, the metabolic flux, and the impact from the technological platforms and methodology utilized in the analysis. Therefore, it is of high relevance to establish the right approaches, and to have adequate experimental designs and reliable protocols; consequently, this will allow for high reproducibility and relevant biological data.

Traditionally, there have been two major approaches used in metabolomics: untargeted and targeted (it is possible to include the semi-targeted analysis as an additional approach). These methods differ in numerous aspects such as the complexity of the sample preparation, the experimental precision, the range of features (metabolites) detected, and the quantification level (relative vs. absolute). Those characteristics lead to setting specific objectives with each approach, such as generating a hypothesis or testing a formed one. Nevertheless, the most relevant difference between both strategies is related to the identification capacity and the structural elucidation of the detected features [22].

The main outcome of the untargeted analysis relies on detecting as many features as possible in a sample through platforms with high precision analytical capabilities. This approach is focused mainly on identifying patterns (fingerprints) or differential metabolic profiles between sample groups. Nevertheless, many of the detected features are unknown, although their identification is possible through the use of in silico libraries or experimental research which must be confirmed using analytical chemistry. Thus, the chemical identification and the structural elucidation are essential for a proper biological interpretation. Nevertheless, the identification still continues to be a challenge due to analytical and methodological reasons. Knowledge of at least the compound class of the metabolites to explore may be useful to help choose the analytical platform and the most suitable method to find the targeted compounds.

As a counterpart, in the semi-targeted and targeted analyses, the chemical compounds studied are previously characterized and annotated. Thus, the analysis focuses on providing high precision, selectivity, and quantification. The semi-targeted approach, also known as “metabolomic profiling”, focuses on an a priori selection of a pathway or a set of metabolites related to it and analyzes their evolution. The previous knowledge of the metabolites included in the analysis has the advantage of providing the ability to understand the biological processes and pathways that are involved immediately after data processing. Therefore, when there is an absolute quantification of a particular pathway it is known as “metabolic targeting” (targeted approach). Thus, these approaches are useful in hypothesis-driven studies in which the goal is to quantify compounds, such as in determining concentration and/or bioavailability, or in studying the metabolism of specific dietary compounds. Nonetheless, the principal limitation of these approaches is due to the poor availability of purified standards to calibrate and validate the assays.

## 3. Analytical Technologies Update

Due to the complexity of the metabolome, today, it is technologically impossible to determine, to quantify, and to identify each of the metabolites present in a biological sample. Thus, it is necessary to use different instruments or platforms, either separately or combined, for such analysis. However, each platform has its advantages and limitations regarding its main capacities, such as the specificity and sensitivity. Among the techniques used in metabolomics studies, it is possible to find: gas chromatography-mass spectrometry (GC-MS), capillary electrophoresis coupled with mass spectrometry (CE-MS), liquid chromatography-mass spectrometry (LC-MS), and liquid and electrochemical chromatography-mass spectrometry (LC-EC-MS). Likewise, nuclear magnetic resonance spectroscopy (NMR), liquid chromatography NMR (LC-NMR), direct infusion mass spectrometry (DIMS), Fourier Transformed Infrared spectroscopy (FT-IR,) and Raman spectroscopy can be used.

However, NMR and MS coupled to the various chromatographic variants (high-performance liquid chromatography (HPLC), GC, EC and LC) are currently the most widely used platforms in nutrimetabolomics studies for optimizing the results. Since the 1970s, these platforms have been used to investigate a series of pathological processes and biological mechanisms using metabolic profiling. They are going to be briefly reviewed in the following section.

### 3.1. Mass Spectrometry

MS provides information related to the molecular mass of the analyzed compounds. Furthermore, the structural information acquired allows the detection or quantification of the concentration. The principle of the method is based on analysis of previously generated ions, which are commonly separated using chromatography to reduce the high complexity of the sample; i.e., Ultra Performance-Liquid Chromatography (UPLC), GC, or EC. These ions are thrown into an analyzer and separated according to their mass/charge (*m*/*z*) relationship by applying electromagnetic fields or simply by determining the time of arrival at a detector. Upon reaching the detector, the ions produce an electrical signal that is processed, amplified, and sent to a computer. The result obtained is called the mass spectrum, and the ionic abundances therein obtained, according to the *m*/*z* of the ions detected, are represented and define the signature of the original molecule.

The selection of the MS instrument is based on both the chromatographic system and the aims pursued. For example, GC-MS provides a high peak capacity, and an excellent repeatability of the retention times. Moreover, the easy access to compound libraries allows the identification of compounds without the need to use internal standards. However, due to the requirement of chemical derivatization of the sample, it is impossible to save it for further analysis. The GC-MS permits the separation of volatile compounds, such as fatty acids and organic acids. Otherwise, the use of LC-MS analysis allows the detection of a broader range of metabolites when using different types of columns, thus, the detected compounds can be of low or high molecular weight and with hydrophilic or hydrophobic characteristics. Furthermore, DIMS has been utilized as a reliable tool for a fast broad coverage of molecules [23].

The main objective pursued in our experiments will lead us to the selection of the MS instrument. Global metabolic profiling is characterized by the need to determine the elemental composition of specific features. Therefore, the use of MS with high-resolution accurate mass determination (HRAM) together with the capability of tandem mass measurements for structural characterization might be the right solution. This is because the high-resolution helps in determining not just the elemental composition but the isotopic ratios of the features detected. Nevertheless, the HRAM MS system’s weakness is related to the poor linear dynamic range; therefore, the quantitative properties are not as good as low-resolution quadrupole MS instruments. Hence, for qualitative analyses, low-resolution mass spectrometers such as quadrupole systems are often a good solution, with the triple quadrupole systems for LC-MS and the single quadrupole systems for GC-MS [24]. We suggest the work of Pohö et al. [24] in which the author deeply discusses different topics regarding MS, such as ionization modes, interfaces, and instrumentation.

### 3.2. Nuclear Magnetic Resonance Spectroscopy

Spectroscopy is the study of the interaction of electromagnetic radiation and matter, with the absorption or emission of radiant energy. NMR is based on the use of the nuclear magnetic resonance phenomenon to study the physical, chemical, and biological properties of matter. Therefore, the NMR technique is highly efficient and useful to explore the structure and dynamics of molecules in a solution. NMR is characterized by its reproducibility, ease of the sample pre-treatment, for having no dependence on the separation of analytes before analysis, and for being a non-destructive technique; therefore, the samples may be used for further analysis. By contrast, the low sensitivity (levels are around the range of 10 µM) represents a significant disadvantage. Nevertheless, the emergence of cryoprobes has improved the levels of sensitivity and increased the dynamic range, and furthermore, allowing the measurement in the millimolar to nanomolar ranges. Nonetheless, the use of labeled compounds has refined this method and depending on the atomic nuclei being targeted by the applied magnetic field, different types of metabolomic data are generated. For example, in samples of biological origin, the most commonly targeted nucleus is hydrogen (^1^H-NMR). Furthermore, other atoms such as carbon (^13^C-NMR) and phosphorus (^31^P-NMR) have been utilized to obtain information of specific metabolite types [25]. Additionally, dynamic nuclear polarization has arisen as one of the most efficient developments to enhance the NMR sensitivity [26]. Consequently, NMR has been shown to be a good solution for metabolic fingerprinting in human studies [27,28].

As a complement to the present review, Alonso et al. [29] recently presented an article in which they update the current knowledge regarding the most commonly used analytical methods in metabolomics. Their work presented different tools to handle the metabolomics data processing through all its stages. Furthermore, Emwas [30] reviewed the literature and presented an interesting resource regarding the strengths and weaknesses of MS and NMR, with a particular focus on metabolomics research.

## 4. Study of Plant-Based Products through the Use of Metabolomics

Nowadays, consumer interest in purchasing “natural and healthy” products has increased due to the well-documented idea that diet has a significant impact on human health and well-being [31,32]. Therefore, manufacturers are looking for solutions in order to improve their products regarding quality, safety, and nutritional properties. Thus, nutrimetabolomics has been shown to be an excellent tool to assess food quality, as it can be used to understand the influence of plant-based product BACs derived from different cultivars, geographical origin, weather and climatic differences, storage, and industrial processing. Additionally, is well-known that the BAC content in fruits or vegetables, such as antioxidants, may be influenced by variety, environment, and agronomic conditions, and these factors may impact its organoleptic properties. For example, carrots are recognized for their BACs associated with anti-inflammatory, antioxidant, antimicrobial, antiviral, and anticancer properties [33]. In this regard, Tomassini et al. [34] studied different ready-to-drink carrot juices from the same cultivar, cultivated by the same methods and processed in the same factory, using an H-NMR approach. They found pedoclimatic-related biochemical differences, mainly based on amino acids and organic acids.

Likewise, Karlund et al. [35] investigated the differences among three different cultivars of both organically and conventionally grown strawberries. Their approach included an untargeted analysis using Liquid Chromatography-Quadrupole Time of Flight-Analysis with Electrospray Ionization-Mass Spectroscopy (LC-QToF-ESI-MS) plus the addition of sensory data from a panel of experts. The authors found that the main differences were related to the cultivar. Moreover, they described that flavonoids and condensed and hydrolysable tannins determined the orosensory properties, and fatty acids contributed to the odor attributes of strawberry. Afterward, Akhatou et al. [36] studied the impact of agronomic conditions in three selected strawberry cultivars classified according to their sensitivity to environmental stress. Their untargeted GC-MS analysis revealed significant alterations among the varieties utilized. These main differences were related to sugars (fructose, glucose), organic acids (malic acid, citric acid), and amino acids (alanine, threonine, aspartic acid), among others.

Vallverdú-Queralt et al. [16] developed a comprehensive biochemical study for determining the differences between conventional and organic tomato-based products (ketchup). Interestingly, the use of an untargeted quadrupole time-of-flight mass spectrometry (QToF-MS) metabolomics approach revealed that the agronomic environment produced alterations in the antioxidant capacity, and in the content of phenolic compounds and other metabolites in ketchup. Those differences were associated with a higher content of antioxidant microconstituents in the organic products. In another work related to the study of tomatoes, Mazzei et al. [37] utilized High-Resolution Magic-Angle Spinning Nuclear Magnetic Resonance (HRMAS NMR) spectroscopy for investigating specific changes in the plant health. Their outcomes were associated with using different secondary metabolites from *Trichoderma* fungi in *Solanum Lycopersicum* treatment. After 15 days of treatment with 6-pentyl*-2H*-pyran-2-one and harzianic acid, the tomato leaves showed a significant enhancement of acetylcholine and γ-aminobutyric acid, which impacted the germination rates and the seedling fresh weight. The use of HRMAS NMR proved to be a rapid and reliable technique for evaluating those specific variations.

In another study, 14 modern apple cultivars were studied in search of new tools to discriminate among closely related apple species. Eisemann et al. [38] applied an untargeted ^1^H-NMR approach, finding that the analysis of peel or pulp extracts represents an efficient tool to differentiate between closely related species and to assess quality control; however, it may not be a priori suitable for use in more advanced applications.

Meanwhile, in a study conducted by Khalil [39], 13 date palm fruit varieties were profiled, focusing on their volatile constituents, and looking for the responsible compounds of their aroma. The analysis revealed 89 metabolites; the phenylpropanoid derivatives were shown to be the major components of date fruit aroma. With the support of a multivariate data analysis, the authors reported 2,3-butanediol, hexanal, hexanol, and cinnamaldehyde as the most discriminant compounds among the different varieties. Diboun et al. [40] designed an ambitious approach for understanding the date fruit biology. They collected 109 unique date palm fruit varieties from 14 countries and assessed their metabolomes in two different laboratories with similar analytical techniques. The authors indicated the importance of date ripening in the variation of the metabolome. Indeed, they found three distinct metabolic stages which correspond to the different biological stages of date ripening. In brief, during the first stage, there was an increase in the production of energy, regulatory hormones, amines and polyamines, tannins, sucrose, and the antioxidant activity. The next phase was characterized by alterations in the phenylpropanoid secondary metabolism, gene expression, and phospholipid metabolism. The final stage was recognized by sugar dehydration activity and an increase of volatile metabolite synthesis.

Regarding the study of ripening, Steingass et al. [41] investigated ripening-dependent changes in the volatile pineapple compounds via headspace solid phase microextraction, analyzed by 2D GC-MS. This approach identified the rapid distinction between premature green-ripe pineapples, postharvest-ripened sea-freighted fruits, and green-ripe fruits at the end of their commercial shelf life.

The use of metabolomics in the study of the impacts of geographical and other agronomic factors on the quality and content of food BAC is rising, and it promises future novel approaches. Several authors are focusing on this route due to the importance of identifying the impacts of the different determinants in agriculture. In the search for answers, Lee et al. [42] profiled two different species of curcuma growth at various locations. Their approach included targeted GC/ToF and UPLC/QToF-MS techniques. The authors found that the concentration of curcuminoids, the representative BACs of curcuma, was different between the samples according to species or geographical origin.

In another approach, Millan et al. [43] studied the differences of grapes according to their varietal type. The authors used an LC-QToF-based targeted analysis and focused on studying the phytosterols family in six different varieties of Rioja grapes. Regarding technical advances, they reported that gas phase ionization achieved the best solution via atmospheric pressure chemical ionization in positive mode. Furthermore, analysis with electrospray (ESI) was needed for phytosterol derivatives confirmation. Overall, the use of LC-QToF required informatics and statistical tools for data processing for the correct identification of metabolites according to their variety. However, further work is needed for a definite confirmation of the features detected in this study.

Other authors utilized an LC-MS method to extract information from different families of phenolic compounds in Spanish wines [44]. They included different statistical methods such as Principal Component Analysis (PCA), Partial Least Squares Regression Discriminant Analysis, and obtained classification rates above 93%. This approach let them classify Penedes, Ribera del Duero, and Rioja wines according to their fingerprints. Moreover, after obtaining their profiles, it was possible to discriminate the geographic controlled appellations based on ten metabolites, i.e., gallic acid for Penedes, *trans*-coumaroyl tartaric and *trans*-caffeoyl tartaric for Rioja, and myricetin for Ribera del Duero [44].

Similarly, in 2015, Narduzzi et al. [45] compared four American wild grapes with different *Vitis vinifera* cultivars through an untargeted LC-MS approach. The primary results obtained from this research were related to a more complex content of anthocyanins and stilbenoids in the American grapes and a higher procyanidin accumulation in the vinifera skin and seeds. Moreover, there was a lack of accumulation of pleasing aroma precursors in American grapes, whereas they are common in vinifera grapes. Finally, they reported for the first time an accumulation of hydrolysable tannins and their precursors in the skin of the American grapes. This research provided valuable data to improve the design of new breeding programs, thus, making it possible to avoid the retainment of undesirable features in the chemical phenotype of the offspring.

Additionally, besides the biotic stress of plants, there are several abiotic factors, such as drought, heat, salinity, or nutrient deficiency that may impact plant development and reduce yields in crop fields. In this regard, Savoi et al. [46] studied the prolonged drought effect on white grapevines using LC-MS. Their main results revealed that grapevine berries respond to drought by modulating several secondary metabolic pathways, and particularly, by stimulating the production of phenylpropanoids, the carotenoid zeaxanthin, and volatile organic compounds such as monoterpenes, with potential effects on grape and wine antioxidant potential, composition, and sensorial features. Hochberg et al. [47] investigated the variability in berry metabolism under scarcity of irrigation in the Shiraz and Cabernet Sauvignon varieties. The study was conducted using GC-MS and LC-MS untargeted approaches on the berry skin at different developmental stages. In response to water deficit, Cabernet Sauvignon showed milder metabolic alterations of berry skin primary metabolites; mainly, a diminishment of amino acids and tricarboxylic acids cycle (TCA) cycle intermediates. Nevertheless, as a response to water deficit, several stress-related metabolites were accumulated in the Shiraz. Finally, the polyphenol metabolism responded differently according to each specific cultivar.

The organic acids present in grapes are sensitive to temperature conditions. In this regard, Sweetman et al. [48] reported about the manipulation of temperature conditions to capture the warming-driven reduction of malate content in Shiraz berries using a GC-MS approach. As a result, the elevation of maximum temperatures during pre-véirason and ripening stages reduced the malate content. However, an increase of the minimum temperatures did not diminish the malate content. The authors suggested an enhancement of the anaplerotic capacity of the TCA cycle and to confront the decreased cytosolic pH. This is supported by the increase of NAD-dependent malic enzyme activity and the reduction of the pyruvate carboxylase and pyruvate kinase activities, as well as the accumulation of various amino acids and γ-aminobutyric acid.

Obata et al. [13] focused on studying the impact of drought and heat on maize leaves, using an untargeted GC-MS approach. Drought stress induced an accumulation of different amino acids, such as isoleucine, valine, threonine, and 4-aminobutanoate. Moreover, two photorespiratory amino acids, glycine and serine, and myoinositol, were also accumulated under drought. However, the combined response of drought and heat stress evoked few specific changes. Altogether, both the photorespiration and raffinose family oligosaccharide metabolism affected the grain yield during drought. Moreover, the authors reported several metabolites as potential metabolic markers for the cultivation of abiotic stress-tolerant maize.

Storage conditions has been shown to impact plant-based food quality. For example, peaches are highly perishable at ambient conditions; however, cold storage helps prevent the fruit decay. Nonetheless, this process alters the physiological properties of the fruit. Lauxmann et al. [49] investigated the primary metabolic differences between diverse conditions of peach storage using a GC-MS platform. They described dramatic changes in the levels of galactinol and raffinose, while amino acid precursors of the phenylpropanoid pathway and the polyamine, putrescine, were also modified due to the stress treatments. Indeed, these findings might provide new knowledge related to plant breeding.

Additionally, in an extensive review from Kusano et al. [50], they summarized the current knowledge on metabolites contained in rice determined using GC-MS, LC-MS, and CE-MS. Briefly, they discussed the presence of various types of metabolites, including primary metabolites, volatiles, phytohormones, lipids, and other secondary metabolites such as phenolics, flavonols, carotenoids, and alkaloids. Their emphasis on the importance of the identification of the chemical diversity of different types of rice is based on the improvement of nutrition quality and an accurate identification of varieties. Therefore, it is necessary to understand the main differences within plant-based foods related to agricultural factors and to take advantage of them in order to improve their nutritional quality.

Metabolomics workflows are being enhanced for different applications such as food authentication. Recent work from Cuadros-Rodriguez et al. [15] reviewed the literature and found that there are more than 50 scientific articles regarding the use of chromatographic methodologies utilized in food authentication. However, although the application of the different analytical and statistical techniques work well in-house, there is a necessity to improve the transferability of the protocols and to let third-party labs reproduce the experiments.

In a recent study, Jandrić et al. [51] worked on identifying the possible adulteration of different fruit juices with cheaper alternatives; for this objective, they utilized an untargeted metabolite fingerprinting approach using an UPLC-QToF-MS platform. Their first results allowed them to identify 21 metabolites that were useful for recognizing adulterated juices down to a level of 1% adulteration. Furthermore, they went one step further through the use of a targeted approach and optimized the method to detect the adulteration at 1%.

Moreover, Fotakis et al. [14] used NMR metabolic fingerprinting to investigate different Greek grape marc spirits, to establish their authenticity. This broad approach included 57 different spirits produced in 2009; the authors aimed to alleviate the contribution of the vintage year to their metabolic signature. An extensive use of chemometrics allowed the authors to benefit from the metabolomics data obtained, i.e., PCA helped to delineate the provenance of the samples and discriminate among subgroups. Furthermore, the authors provided a classification based on the grape cultivar which is addressed by a “varietal evolution”. The use of supervised analyses helped to explain the metabolic variations related to the “terroir” influence. Moreover, the use of supervised data classification methods in samples from the same origin revealed the principal metabolites for the genotypic classification. The application of this type of model reduces the influence of the geographical origin and the impact of the viticultural practices.

Also, this scheme has shown to be effective regarding plant-based brand-protected products (appellations or geographical indications). For example, Caligiani et al. [52] used a ^1^H-NMR metabolomics approach to improve the identification of the Italian hazelnut cultivar “Tonda Gentile Tribobata (TGT)”. The identification was primarily based on morphological characteristics; thus, the metabolomics approach brought an objective analytical method for authentication. As a result, the classification models allowed the authors to distinguish TGT from other types based on a metabolic profile which included trigonelline, amino acids, and one unidentified *orto*-disubstituted aromatic compound. Later on, Locatelli et al. [53] understood that food processing, such as roasting, might modify the chemical profile of the hazelnuts, compromising, as a consequence, its identification. Their approach included different analyses including the determination of phenol content, antioxidant activity, polyphenolic profile, and protein fingerprinting, among others. The developed methodology, in addition to an extensive chemometric analysis, yielded preliminary conclusions that showed that despite the roasting process, it is still possible to identify hazelnuts according to their origin.

The study of the effects of industrial processing in plant-based products is another interesting application of metabolomics. Lopez-Sanchez et al. [54] applied a complex metabolomics approach, which included the use of HPLC equipped with both a photodiode array and fluorescence detectors for vitamins, ^1^H-NMR for polar metabolites, accurate mass LC-QToF-MS for semi-polar metabolites, LC-multiple reaction monitoring (MRM) for oxylipins, and headspace GC-MS for volatile compounds. Their study focused on broccoli, tomato, and carrot purees, which have been shown to be severely affected by blending and thermal treatment. Notably, the blending-heating order was proven to affect the metabolites present in the puree, particularly, potential health-related phytochemicals. These results are relevant for the industry to go further in the study of processing steps and their impact on the final products and, as a result, to manufacture products with a better nutritional quality.

During the last few years, there has been an enhancement in metabolomics platforms regarding their sensitivity and it is now possible to detect a broader range of compounds. Therefore, other research lines are arising; one of them is focused on studying the secondary metabolites, also known as phytonutrients, of plant-based foods and extracts. Phytonutrients are implicated in a range of activities which may impact its host at the metabolic level; i.e., reducing oxidative stress, regulating signal transduction pathways, transcription factor function, gene expression, enzyme activity, and other cellular and subcellular processes [55,56]. Therefore, these BACs might moderate the inflammatory and oxidative responses associated with the onset and development of NCCDs [12]. It is important to study the whole set of metabolites rather than each one independently, as this will help to understand the entire mechanism in which BACs provide their benefits.

In this regard, Paudel et al. [12] investigated the bioactivity of phytonutrients in a black raspberry fruit extract. Using an untargeted H-NMR-based metabolomic approach, they reported cyanidin 3-rutinoside and cyanidin 3-xylosylrutinoside as the main contributors to the extract bioactivity. Nevertheless, salicylic acid derivatives (e.g., salicylic acid glucosyl ester), quercetin 3-glucoside, quercetin 3-rutinoside, *p*-coumaric acid, epicatechin, methyl ellagic acid derivatives (e.g., methyl ellagic acetyl pentose), and citric acid derivatives are responsible for the antiproliferative activity of the berry extracts [12].

In 2013, Beckmann et al. [57] studied BACs associated with whole grain sourdough rye bread. In their intervention study, the participants consumed 48-g whole grain rye foods per day for four weeks and afterwards they doubled their intake for four weeks. The authors analyzed 24-h urine samples through a flow infusion electrospray MS. They identified the presence of conjugates of the benzoxazinoid lactam 2-hydroxy-1,4-benzoxazin-3-one and hydroxylated phenyl acetamide derivatives, which seem to be potential bioactive alkaloids.

Li et al. [58] recently reported the discovery of a novel compound, oxyphylla A ((*R*)-4-(2-hydroxy-5-methylphenyl)-5-methylhexanoic acid) derived from the fruit *Alpinia oxyphylla*. The authors applied an LC-MS-based multivariate data analysis and a guided fractionation strategy which included metabolomics, pharmacology, and chemometrics for the study. Interestingly, the reported compound showed potential neuroprotective properties against Parkinson’s disease using a primary neuronal Parkinson’s disease model.

Regarding another type of plant-based product, Nakabayashi et al. [59] studied the spears of *Asparagus officinalis*, searching for BACs containing sulfur. After applying a targeted MS approach, they reported asparaptine as a new bioactive metabolite present in asparagus; this metabolite may provide an inhibitory activity against angiotensin-converting enzyme due to its sulfur content. However, this must be corroborated through a Randomized Clinical Trial (RCT).

## 5. Evidence for the Role of BACs in Health and NCCD Prevention

Nowadays, it is still difficult to study the metabolic fate of food and beverages with complex phytochemical ingredients; this is because of the lack of information regarding the specific composition and the identity of the BACs present in food. Besides, the absorption and metabolism of these BACs in humans is highly variable. Moreover, most of the studies have hypothesized that BACs may be absorbed. However, this is probably not correct at all. Nevertheless, the use of modern omics-type technologies presents a more thorough, less biased analytical method, which may provide new insights. In order to maximize the results of metabolomics analysis, it is necessary to use a combination of different analytical methods for the profiling of the small molecules, usually involving NMR spectroscopy or hyphenated chromatography-MS. This must also be accompanied by univariate and multivariate statistical analysis, to make comparisons among the studied groups or controls [60].

The opportunity of quantifying and recognizing the pathways in which metabolites are involved allows us to understand their mechanisms of action and the interactions that may exert their beneficial effects. The use of metabolomics in NCCDs through the development of RCTs is revealing the role of specific BACs. Nonetheless, it is important to comprehend the function of each metabolite in the adequate context. For example, the primary function of glucose is to act as an energy source, but when its presence is increased in urine, it is associated with diabetes [61].

The number of people at risk of developing NCCDs has risen dramatically throughout the world. Thus, the study of the effect of BACs in several risk factors and disease might help to find new strategies to prevent the increase of these prevalent diseases. Among risk factors, obesity is characterized by excessive fat accumulation and is associated with several pathological disorders that may lead to different metabolic diseases. During the last decade, the number of metabolomics studies focused on studying the role of BACs in obesity has increased. Nonetheless, some authors have studied the effect of plant-based products in health through metabolomics approaches without significant results. For example, in different RCTs using a GC-MS platform, Nieman and colleagues examined the effects of a number of plant-based foods, such as chia [62,63], red pepper, and turmeric [64] regarding weight loss and different disease risk factors. However, the authors reported no significant effect related to any of the BACs concerning weight loss or other risk factors such as inflammation or oxidative stress. Schär et al. [65] compared the effect of orange juice (OJ) intake vs. hesperidin or control, in male subjects with cardiovascular risk, through an HPLC-MS targeted approach, concluding that there were no significant differences in cardiovascular disease (CVD) associated metabolites among the groups. It is essential to remember that the inter-individual variations are considered to enhance the experimental designs. This issue can be represented in the ambitious design of Mikkelsen et al. [27], who studied the effects of β-glucans in humans using an untargeted NMR approach. The authors designed a randomized, blinded, cross-over 3-week intervention to study the hypocholesterolemic effects of 3.3 g β-glucan/day from three equally sized but structurally different oat and barley β-glucans. The plasma metabolome did not show any significant change after the intervention. However, the authors reported unique lipoprotein profiles, which were dependent on gender, body mass index (BMI), and diet. Notwithstanding, other authors have reported positive advances.

Tulipani et al. [66] studied the urinary metabolic signature of subjects with metabolic syndrome after 12 weeks of nut consumption through an HPLC-QToF-MS-driven untargeted metabolomics approach. Their analysis revealed 20 potential markers of nut intake, including fatty acid conjugated metabolites, phase II metabolites (particularly of urolithins coming from the metabolism of walnut ellagitannins), microbial-derived phenolic metabolites, and serotonin metabolites. Urolithins may have a potential role as anti-inflammatory agents and therefore, reduce the cardiovascular risk. The confirmation came in a later work [67] in which the authors found that higher levels of urolithin A glucuronide can be found in subjects with less severe metabolic syndrome traits, especially in females.

Llorach et al. [68] developed an analysis comparing the consumption of cocoa with skim milk and a control intervention without cocoa through an LC-MS approach. They included subjects with high risk of CVD and reported a clear separation between samples from the experimental group and the control group after the intervention. This separation was mainly related to 39 compounds linked with the cocoa intake, including alkaloid metabolites, polyphenol host and gut microbial metabolites (hydroxyphenylvalero-lactones and hydroxyphenylvaleric acids), diketopiperazines, and *N*-phenylpropenoyl-l-amino acids. Furthermore, metabolites linked with carnitine metabolism and from the sulfation of tyrosine were decreased after cocoa consumption. Those metabolites may be implied in metabolic disorders related to CVD.

Recently, our research group studied the effect of an intervention with two different OJs containing different amount of flavanones using a Ultra- High Performance Liquid Chromatography-Mass Spectroscopy (UHPLC-MS) untargeted approach. The primary objective of our research was to identify possible biomarkers of OJ consumption and its relationship with NCCDs risk factors such as oxidative stress and inflammation. The metabolomics data obtained permitted us to design a panel of biomarkers, which included betonicine, stachydrine, methyl glucopyranoside (α + β), dihydroferulic acid, and galactonate, to identify OJ intake. Moreover, the metabolites associated with the OJ consumption were related to the shift of different inflammatory and oxidative stress-related metabolites [69]. However, further analyses in a bigger cohort are needed to validate the results that were reported.

Diabetes is one of the most common NCCDs globally; in 2014, 387 million people already had diabetes, and the number is expected to rise to 592 million by 2035 [1]. People with diabetes present numerous deranged pathways, particularly those related to energetic fuels. Therefore, the possibility of measuring small molecules may provide a deep insight into the molecular pathology of diabetes and its response to the presence of BACs. In this regard, Al-Zuady et al. [70] studied the antidiabetic and antioxidant activities of different *Melicope Lunu-ankenda* (ML) ethanolic extracts through NMR and UHPLC-MS/MS techniques. After confirming the protective properties of ML, they identified the BACs responsible which included: isorhamnetin, skimmianine, scopoletin, and melicarpinone.

Furthermore, colorectal cancer (CRC) is the third leading cancer cause of mortality in the world, and there are several strategies to study its prevention and to understand the underlying mechanisms of the NCCD. In this context, Li et al. [71] studied the disease in mice harboring adenomatous polyposis coli gene mutation-induced colon cancer using a UPLC-ESI-QToF-MS metabolomics analysis. First, they reported four uremic toxins (cresol sulfate, cresol glucuronide, indoxyl sulfate, and phenyl sulfate) generated in the microbiota and associated with pro-inflammatory profiles. After an intervention in the Adenomatous polyposis coli (*Apc*)^min/+^ mice with nutmeg, they reported a reduction of the uremic toxins and a decrease in intestinal tumorigenesis. Furthermore, the pro-inflammatory profiles were reduced, showing a possible relationship between the uremic toxins and the development of tumorigenesis; although, a therapeutic value against gastrointestinal cancer had been demonstrated.

Nuñez-Sanchez et al. [72] studied the effect of a pomegranate extract in urine and colon tissues from 33 CRC patients. The subjects consumed 450 mg of either a low or high punicalagin content extract before surgery. The authors used an UPLC-ESI-QToF-MS/MS approach and reported an accumulation of several ellagic acid derivatives and urolithins, which are BACs produced by the gut microbiota. These BACs may exert potential cancer chemoprotective effects.

Similarly, Guertin et al. [73] investigated the relationship between coffee consumption and CRC, through an untargeted approach using UHPLC-tandem MS and GC-MS. Firstly, the authors aimed to identify metabolites associated with coffee consumption and secondly, examined these metabolites in regard to CRC. Their study included 498 subjects, 251 cases, and 247 controls, and they described 29 metabolites associated with coffee consumption, of which theophylline, caffeine, and paraxanthine were inversely linked to CRC.

Finally, Pan et al. [74] studied the effect of freeze-dried blackberries on CRC. Their approach included the analysis of urine and plasma samples using UHPLC-MS/MS (positive and negative mode) and GC-MS. They reported the shift of 40 metabolites, with the most relevant change being the increase of 4-methylcatechol sulfate in both urine and plasma after the blackberry intervention; furthermore, this increase was correlated with a higher level of the apoptotic marker (TUNEL). Moreover, the increase of benzoate was associated with enhanced levels of amino acid metabolites, suggesting that blackberry consumption may modulate some energy generating pathways.

## 6. Conclusions

The use of metabolomics in nutritional research is imminent. The existing platforms and systems used to analyze biological samples from food and nutrition studies are highly evolved and now allow us to answer several research questions that have arisen during the last few years. However, it is important to understand that not only one platform, such as MS or NMR, will provide the entire information from the metabolome; it is necessary to combine them to obtain reliable data with a valid biological interpretation. Furthermore, there are several methodological and technical enhancements to take into account. Firstly, it is necessary to share data and homologate terms among the metabolomics community. In this way, we will be able to increase reproducibility, currently the primary goal. Secondly, during the design of new clinical trials, it is of great importance to take into account the inter-individual variability to reduce bias.

Nutrimetabolomics studies related to plant-based foods are also arising. The application of several strategies is right around the corner and will permit the study of the entire metabolome of fruits, vegetables, and seeds, among others. In the present review, we had demonstrated that metabolomics is showing promising advances regarding the assessment of food quality and authentication. Moreover, the implementation of metabolomic analysis demonstrated its reliability in the study of cultivars and the many factors that could impact its development such as variety, geographical origin, weather and climatic variations, storage, and abiotic factors, among others.

Furthermore, the use of metabolic profiling is generating valuable data regarding BACs and its presence in plant-based food products. However, several RCTs utilizing BACs for prevention of disease have shown no significant effect. Albeit, it is important to stress that it is necessary to increase the population included in future studies and to take into account the inter-individual variation, when designing new RCTs.

Notwithstanding, the use of nuts or OJ has revealed significant effects in several metabolic risk factors. Likewise, the evidence of the impact of BACs on cancer is promising, with studies including blackberries, coffee, nutmeg, and pomegranate supporting this. An in-depth knowledge of the small molecules present in plant-based foods will help to understand the mechanisms of their beneficial effects on health and the prevention of NCCD.

## Abbreviations

APC	Adenomatous polyposis coli
BAC	Bioactive compounds
CRC	Colorectal cancer
DIMS	Direct infusion mass spectrometry
EC	Electrochemical
ESI	Electrospray ionization
FT-IR	Fourier transformed infrared spectroscopy
GC-MS	Gas chromatography-mass spectrometry
HRMAS NMR	High-resolution magic-angle-spinning nuclear magnetic resonance
LC-MS	Liquid chromatography-mass spectrometry
ML	Melicope lunu-ankenda
NCCD	Non-communicable diseases
NMR	Nuclear magnetic resonance
OJ	Orange juice
QToF	Quadrupole time-of-flight
RCT	Randomized clinical trial
UHPLC	Ultra-high performance liquid chromatography
UPLC	Ultra-performance liquid chromatography
TGT	Tonda Gentile Tribobata
TOF	Time-of-flight
